# On Structural Entropy and Spatial Filling Factor Analysis of Colonoscopy Pictures

**DOI:** 10.3390/e21030256

**Published:** 2019-03-06

**Authors:** Szilvia Nagy, Brigita Sziová, János Pipek

**Affiliations:** 1 Széchenyi István University, Egyetem tér 1, H-9026 Gyor, Hungary; 2 Budapest University of Technology and Economics, Budafoki út 8, H-1111 Budapest, Hungary

**Keywords:** computer aided diagnostics, colonoscopy, Rényi entropies, structural entropy, spatial filling factor

## Abstract

Colonoscopy is the standard device for diagnosing colorectal cancer, which develops from little lesions on the bowel wall called polyps. The Rényi entropies-based structural entropy and spatial filling factor are two scale- and resolution-independent quantities that characterize the shape of a probability distribution with the help of characteristic curves of the structural entropy–spatial filling factor map. This alternative definition of structural entropy is easy to calculate, independent of the image resolution, and does not require the calculation of neighbor statistics, unlike the other graph-based structural entropies.The distant goal of this study was to help computer aided diagnosis in finding colorectal polyps by making the Rényi entropy based structural entropy more understood. The direct goal was to determine characteristic curves that can differentiate between polyps and other structure on the picture. After analyzing the distribution of colonoscopy picture color channels, the typical structures were modeled with simple geometrical functions and the structural entropy–spatial filling factor characteristic curves were determined for these model structures for various parameter sets. A colonoscopy image analying method, i.e., the line- or column-wise scanning of the picture, was also tested, with satisfactory matching of the characteristic curve and the image.

## 1. Introduction

Colorectal cancer develops from colorectal polyps. The detection of the colorectal polyps is mostly carried out by special endoscopes, called colonoscopes [1,2]. These devices possess not only the image acquiring equipment with light source, but also forceps, needle, laser scalpel, or loop instrument for removing polyps or tissue samples for biopsy. Beside normal, white light pictures, some of the endoscopes can take narrow band images (NBI), which emphasize the blood vessels and the shadows, as can be seen in [3,4,5], which help find unusual vein patterns that are typical in the case of malignant polyps. In many cases, indigo carmine [4] or other food dyes of bluish hue can be sprayed beside the usual cleansing water to make the pits and valleys of the bowel wall more visible (chromoendoscopy). In addition, magnifying endoscopy is becoming more and more common to detect the fine scale patterns of the surface [4]. Virtual endoscopy [6,7] is a computer tomography based alternative for the optical endoscopy. Capsule endoscopy was developed with the goal of decreasing the discomfort of the patients. It is a small capsule with two cameras and light sources at both ends. It can be swallowed, and travels through the bowel [8]. Unfortunately, it is generally less effective in finding polyps than the classical endoscopy, and cannot perform any operations, as it is a passive device.

Although colonoscopy is considered to be the most effective way of cancer screening [1], it still has a non-negligible miss rate [9], which has not decreased much, even though the equipment has improved over time [10]. A colorectal polyp can be missed for the following reasons. First, if the polyps are small, then they are much harder to find [9], even though curvelet-based methods exist that can improve the diagnosis probability [11]. In addition, usually, the better quality the picture is, the lower the risk of missing a small polyp. Moreover, although before endoscopy sections, the bowel is theoretically purified, impurities often occur [12], mostly in the form of yellowish liquid or solid pieces. These impurities can be removed by spraying water on the given surface segment from the endoscope. The next reason is that the bowel has continuously moving, shiny, pink walls, which sometimes (despite the inflation) fold over polyps. The last factor is fatigue: after a couple of seconds of watching the screen of the endoscopes, the eyes and brain of the gastroenterologist gets used to the environment, and after a longer time of watching they often get tired; this is true not only in the case of inexperienced medical staff [9,10]. This last factor was the reason for thinking about developing a computer aided diagnosis protocol. Computer aided diagnosis is not meant to substitute the human medical expert, only to draw their attentions to certain points, in a way that does not disturb other aspects of the diagnosis.

Image processing tools can improve the diagnosis performance; however, colonoscopes are not developed for machine processing, no matter if it is a conventional or capsule version. Usually, live video signal arrives from the endoscope, which has quite low bit rate, hence the small resolution and/or large compression ratio of the pictures. This means that, even though the video signal seems to be of good or at least acceptable quality for the human eye, the individual pictures have large distortion and many compression artifacts. Since the bowel walls move continuously, usually the pictures are blurred. Impurities are often present, and, even if they are removed by water, this water together with the native liquids makes the bowel walls shiny, and the thus arising reflections make image processing more complicated. Even though chromoendoscopy or NBI can make the color spectrum a bit more stretched, in a common endoscopy picture, the colors are mostly only shades of pink.

In medicine, to determine whether a lesion is benign or potentially malignant, a sample is taken from the tissue for biopsy. As fatigue might also influence the working efficiency of a pathologist, there are possibilities to introduce computer aided diagnosis to this point, too. This branch is investigated by multiple research groups. Kayser and his group applied the neighborhood relations based structural entropy for this purpose [13]; dos Santos and his group used sample entropy [14]; Ribeiro and his fellow researchers proposed curvelet and fractal analysis combined with Haralick structure descriptors and various classification methods [15]; Chaddad and his coworkers combined multiple texture characterizing entropy-related quantities and studied their multiresolution behavior [16,17]; and Wang trained neural networks for the picture components derived for both of the applied stains [18]. However, even though these methods might work well for tissue samples or other illnesses [19], they are unfortunately not applicable directly to endoscopy pictures.

Another image classification task related to colorectal polyps or cancer arose after magnifying endoscopy appeared [20]. Kudo and his coworkers, based on biopsy results, found that the pit pattern of the polyp surface can classify the polyps without performing the biopsy, or removing the benign polyps [21]. The classification of polyps based on the Kudo classes [22] is the second branch of image processing based computer aided diagnosis for colonoscopy [23]. However, the task for finding the polyps is also of great interest.

The colorectal polyps can have a wide range of shapes, from lesions depressed into the bowel wall through flat and slightly protruding sessile to pedunculated polyps with expressed stalk. Generally, the roundish polyps—either sessile or pedunculated—are the target for automatic image processing methods. The MICCAI Endoscopic Vision Challenge made several databases and methods available [24,25,26]. Its results are summarized in [27]. The methods collected in [27] include evolutionary algorithms, neural networks, and shape- and lighting-based classical similarity recognition algorithms [25]. For the detailed description of the methods and the results achieved by the research groups that participated in the MICCAI Endoscopic Vision Challenge, we refer to [27]. There are also methods for computer aided classification of lesions from capsule endoscopy images [28,29], but most of the studies work with conventional endoscopes, as they are more widespread.

A fuzzy classification scheme based on the method detailed in [30,31] and summarized in [32] also appeared [33,34]. This proposal uses edge density and statistical parameters, such as the mean, standard deviation or the Rényi entropy based structural entropy, for determining whether a segment of the colonoscopy picture contains polyp.

Entropies are often used for image analysis [19,35,36]. There are two approaches for determining the structural entropy. First, a graph theory based definition was given in [37]. Later, independently a Rényi entropy based structural entropy was also introduced. Although this later structural entropy is first applied in electron structure analysis in [38], its use in image processing is first presented in [39] for characterizing microstructures of the metal electrode materials on semiconductor surfaces. The idea to use it in medical image processing are presented in in [33]. The results are good, well above 90% hit rate for some types of pictures (especially the ones where either the color or the pattern is very different for the polyp and the background, or the polyp has strong, visible contour), while for some other types (e.g., when the polyps are lit too strongly from the side, or when there are image distortions on the pictures due to low resolution or some dark impurities), the miss rate is around 50%, which is of course unacceptable. The false positive rate is always very low, except for some extremely impure cases.

The aim of this paper was not to provide or improve a method for classification of images, but to study a tool that can be used in classification algorithms as one of the parameters related to the shape of the pixel intensity distribution of the picture.

The Rényi entropy based structural entropy is a very simple quantity, easy to calculate, and, together with the spatial filling factor, gives visible information about the shape of the studied distribution. In the following, we discuss some of the most important properties of the roundish colorectal polyps and model their pixel intensity distributions and structural entropy behavior to get characteristic curves that help to understand the reason of differences between image segments with and without polyps and image types.

The remainder of this article is organized as follows. The properties of structural entropy and its use in image analysis are summarized in Section 2. Next, in Section 3, the distribution of bowel picture segments with and without polyps are studied and model structures for reproducing certain aspects of the pictures are introduced, which are required to generate structural entropy–spatial filling factor characteristic curves for the picture segments with polyps, as presented in Section 4. In this section, the dependence of the structural entropy–spatial filling factor curves of both the model surfaces and the real images are studied according to several parameters. Finally, the conclusion is summarized in Section 5. The characteristic curves are collected in Appendix A and Appendix B.

## 2. The Rényi Entropy-Based Structural Entropy

One of the first attempts to describe information was by Hartley, who used a number of yes/no questions to identify an element of a set of possible strings as the information revealed by identifying the string [40]. A couple of years earlier, Nyquist used a very similar formula [41]: both used the logarithm of the number of possible choices to define information content. Shannon wrote his article about the theory of communication in 1948 [42]. He first defined entropy in the information content sense, referring to statistical mechanics and the Gibbs entropy [43] when introducing this quantity. In addition, in quantum mechanics, the entropy of a density distribution was introduced [44], as it was based on the notations used by von Neumann [45]; later, it was named after him. In both cases, the entropy of a probability distribution $\left\{p_{1},p_{2},\ldots ,p_{N}\right\}$ is defined as

(1) $$S\left(p_{1},p_{2},\ldots ,p_{N}\right)=K\sum_{i=1}^{N}p_{i}\log_{a}\frac{1}{p_{i}}.$$

where, both the constant *K* and the basis of the logarithm are freely chosen; however, both in quantum mechanics and in information theory, the constant is generally selected to be one. The basis of the logarithm is 2 in the case of the information theory applications (in this case, the unit of the entropy is Shannon or simply bit), and *e* in the case of physics.

The entropies used in image processing, as well as the quantities originated from the sample or structural entropy usually define the probability distribution corresponding to an image in a rather complex way. Stantchev based the probabilities on the number of connections of a given node in a graph. The entropy from [13,46] calculates probabilities from distances between neighboring vertexes and connections weights; Humeau-Heurtier and her co-workers generalized the sample entropy, which uses probabilities consisting of the ratio of the number of cases when two sample vectors froming a series have sufficiently small distance, and a similar number for their shortened versions [36,47,48,49]. All these methods introduce quite complicated concepts, which are scale-dependent. Of course, scale dependency gives valuable information on the structure, such as in the case of the multiscale entropy [36].

However, there is another concept for characterizing the structure the shape of a picture by entropies. Images have native distributions, their pixel intensities, which can be easily normalized to fulfill the conditions for being a probability distribution, i.e.,

(2) $$\begin{array}{ccc} \sum_{i=1}^{N}p_{i} & = & 1 \end{array}$$

(3) $$\begin{array}{ccc} p_{i} & \geq & 0\;fori=1,2,\ldots ,N, \end{array}$$

if the already non-negative pixel intensities $I_{i}$ are divided by their sum as $p_{i}=I_{i}/\sum_{i}I_{i}$.

In electron structure calculations, instead of the probabilities, the electron density is used; it is also normalized similarly to the probabilities. Although the electron density is usually a continuous function, it can be approximated, or modeled as a distribution over a regular grid, thus the similarity of the electron states and picture pixel intensities can be seen. For measuring how localized is an electron state of a solid, a participation ratio, or delocalization measure, was introduced [50,51] the following way,

(4) $$D=\frac{1}{\sum_{i=1}^{N}p_{i}^{2}}.$$

This quantity tells approximately the number of the higher probability grid points, i.e., the number of grid points the electron density extends to, or, in the case of the pictures, it is the number of the light pixels.

If in the entropy we substitute the real probability distribution by a step distribution that extends to the *D* pixels, we exclude the shape information and keep only the information related to the extension of the distribution. The entropy thus becomes the extension entropy [38]

(5) $$S_{ext}=\log D.$$

This means that, if we subtract $S_{ext}$ from the total Shannon entropy, the remaining part has the information about the shape or the structure of the distribution. Structural entropy was introduced as

(6) $$S_{str}=S_{1}-S_{ext}=S_{1}-\log D.$$

In Ref. [38], Equation (6) uses natural logarithm, and we apply this convention (even though any basis for the logarithm could be used).

Using Shannon’s entropy definition, Alfréd Rényi generalized [52,53] the quantity characterizing the amount of information based on Faddeev’s postulates [54]. His zeroth entropy was Hartley’s information content; the first one was Shannon’s entropy; and the next members of this series are

(7) $$S_{n}=\frac{1}{1-n}\log\sum_{i=1}^{N}p_{i}^{n}.$$

If we study the extension entropy in Equation (5), knowing the Rényi entropy series in Equation (7), we can find that the second Rényi entropy

(8) $$S_{2}=\frac{1}{1-2}\log\sum_{i=1}^{N}p_{i}^{2}=\log\frac{1}{\sum_{i=1}^{N}p_{i}^{2}}.$$

is the extension entropy itself [55].

**Figure 1:**
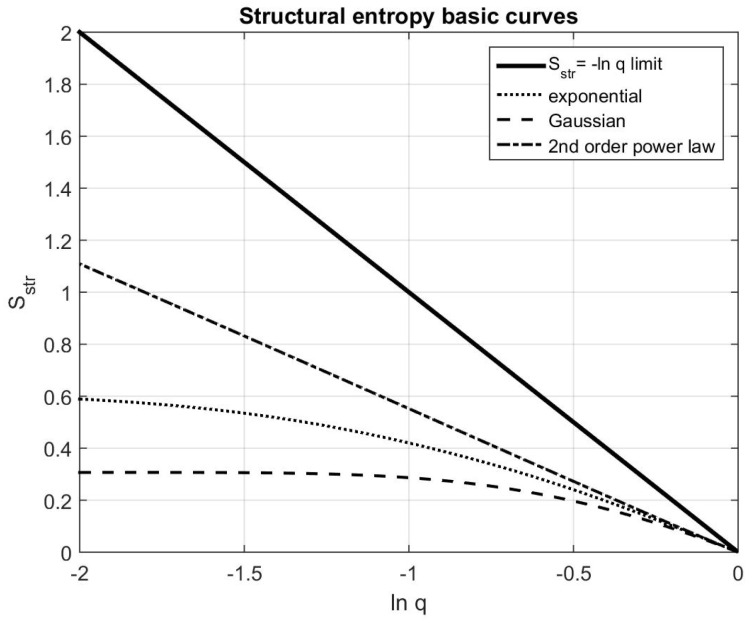
A structural entropy lnq plot showing the limiting line $S_{str}=-\ln q$ in thick continuous line, and the trend lines for the second-order over law, exponential and Gaussian type distributions for two-dimensional case.

Pipek and Varga introduced another quantity that describes the structure of the distribution. If the participation ratio *D* is compared to the total number of grid points (pixels), i.e.,

(9) $$q=\frac{D}{N}$$

is defined, we receive the so-called spatial filling factor, which is a quantity between 0 and 1. Pipek and Varga [38] showed that, if for a distribution of a given shape its structural entropy $S_{str}$ is plotted versus its spatial filling factor *q*, then the point is along a curve that is characteristic for the shape of the distribution. Each type of shape, e.g., Gaussian, exponential or power law distribution has its separate characteristic curve (which is of course different for one-, two-, or three-dimensional distributions). Moreover, in [55], the relation

(10) $$\log q=\log\frac{D}{N}=\log D-\log N=S_{2}-S_{0}$$

is also derived, and it is usual to plot the $S_{str}\left(\ln q\right)$ curves, instead of the originally proposed structural entropy–filling factor plots. Some characteristic curves for the two-dimensional exponential, Gaussian and second-order power law distributions are shown in Figure 1. In addition, the theoretical limit of the structural entropy

(11) $$S_{str}\leq -\ln q$$

is plotted. For the proof of this formula, $S_{str}\geq 0$, the completeness of the allowed domain, as well as the shape of the characteristic curves, we refer to the appendices of [38].

The Rényi entropy based structural entropy and the filling factor is introduced in scanning electron microscope image characterization in [39], and for determining superstructures within a nanostructure in [56]. For characterizing surfaces of electrodes, Bonyár and his coworkers used the structural entropy based localization factor with success [57,58]. Based on these results, we surmised that also colorectal polyps can be identified using their structural entropy versus filling factor plots. The classification of a distribution needs characteristic curves, to which the structural entropy and filling factor point of the distribution can be related. To make the Rényi entropy-based structural entropy applicable for characterizing images or image segments of colonoscopy origin, we need to find possible structures present in such an image, as well as their characteristic lines on the $S_{str}\left(\ln q\right)$ map. The purpose of this study was to determine if there are differences between characteristic curves of images with and without polyps, and if there is a way they might be used for distinguishing the two types of images.

## 3. Results: Simplifying and Modeling the Structures Present on Colonoscopy Images

### 3.1. Across Real Pictures

**Figure 2:**
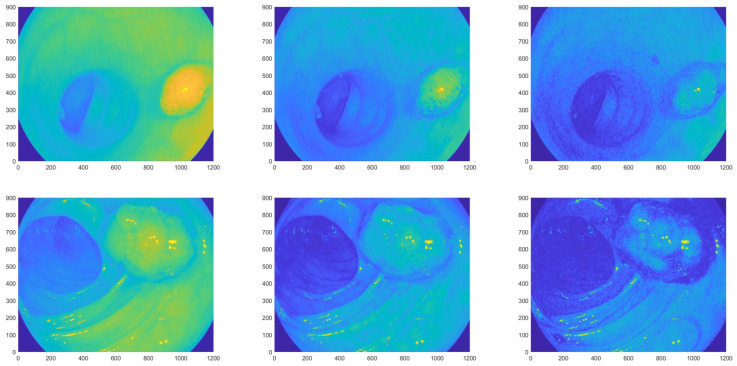
The red, green and blue color channels of pictures 83.tif and 114.tif from database ETIS Larib [24].

We used the database of Etis Larib from the MICCAI Endoscopy Vision Challenge [24] for this study, as their pictures have very high resolution (1225 by 966 pixels), only small black frame, and only very few compression artifacts. The three color channels of two selected images are plotted in Figure 2. (The first one belongs to the well, but not extremely well classifiable group in [33,34], the second to the not too badly classifiable group.) It can be seen that the different color channels emphasize different features of the image: the veins are visible in the green color channel, the shadows can be seen in the blue and red channels and yellowish liquids show in the blue channel.

**Figure 3:**
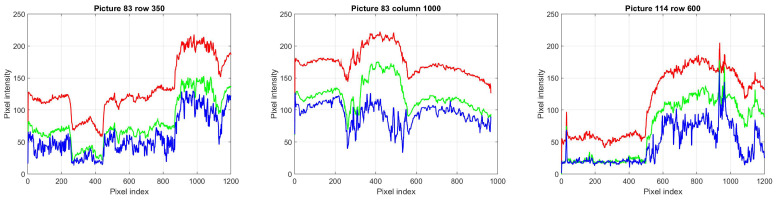
Cross section plots through polyps of Figure 2. The red, green and blue color channels are plotted with red, green and blue, respectively.

In these pictures, the elementary structures seem to be waves and sphere or ellipsoid segments. To understand the behavior of the structural entropy of the different image segments, structural entropy versus filling factor plots of waves with straight or curved wave fronts, as well as of hemispheres are determined and the characteristic lines are given for these structures as a first step. As in these pictures the sphere segments are sitting on the wavy background, the next step would be to plot structural entropy characteristic curves of these superposed structures. To determine the more detailed structure around the polyps, we prepared cross section cuts through the polyps both in both dimensions. Some examples are shown in Figure 3.

**Figure 4:**
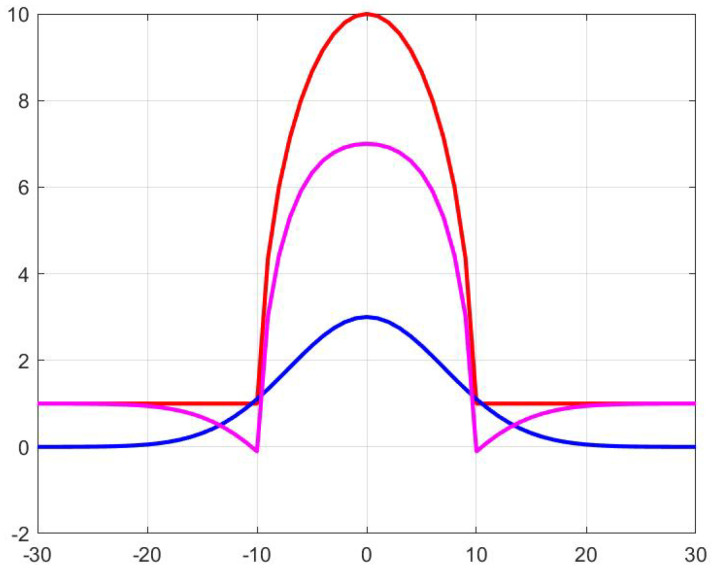
A semicircle (red line), a Gaussian function (blue line) of the same parameters $R_{circle}=\sigma_{Gaussian}$ and their difference (magenta line), which is reminiscent of the shape around the polyp in Figure 3. This kind of function can be used as a simple model the pixel intensity around the polyps.

According to the cross sections, the environment of the polyps can be modeled as if a hard hemisphere would be pressed into an elastic surface, i.e., almost all the polyps had some kind of ditches around them, similar to the function shown in Figure 4. This is of course the shadow around the polyp. After testing some functions to reproduce this structures, we found that, if we subtract Gaussian function of the same standard deviation as the radius of the sphere, the behavior is rather well modeled.

### 3.2. Model Structures for Waves of the Bowel Wall

**Figure 5:**
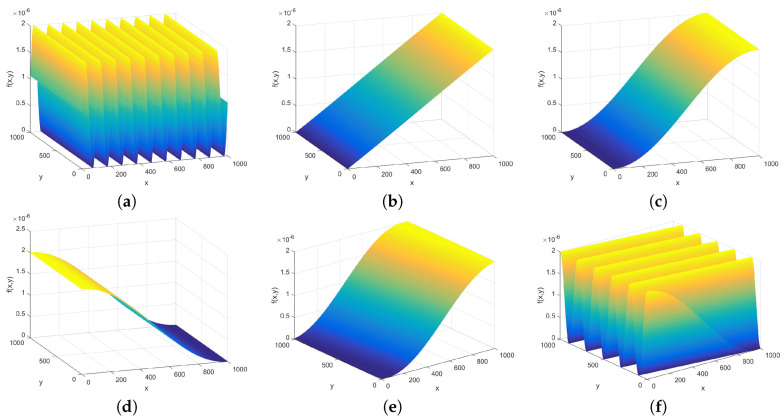
Pictures of straight waves over 1000 by 1000 grid. The limits of the parameter sets are plotted: (**a**,**b**) the largest wavelength and the smallest wavelength used; (**c**,**d**) the minimum and the maximum of the phase shifts; and (**e**,**f**) the minimum and maximum wavefront direction angles.

The bowel wall without polyp forms waves. As a first step, these waves can be modeled as sinusoidal function with straight wave profile over a grid of size N×N, as can be seen in Figure 5. A sinusoidal distribution

(12) $$f\left(x,y\right)=A\cdot \sin\left(\frac{2\pi }{T}x+\varphi \right)$$

has three parameters: the amplitude *A*, the wavelength *T* (frequency 1/T) and the phase φ. In our case, as the distributions are normalized to be a probability distribution, changing the amplitude is out of the question, thus the remaining parameters are frequency and phase shift. An offset or DC term can also be introduced as a parameter to study, and the angle of the wave front, is the distribution, thus becomes:

(13) $$f_{W}\left(x,y\right)=\sin\left(\frac{2\pi }{T_{x}}x+\frac{2\pi }{T_{y}}y+\varphi \right)+B+0.5,$$

with *B* being the offset, and $T_{x}$ and $T_{y}$ the two components of the wavelength. A default offset 0.5 was introduced to fulfill Equation (3). The distribution is normalized according to Equation (2) before calculating the structural entropies in all the cases, thus the normalization step is not mentioned in the further models.

As in most cases only part of a whole period is visible in the studied image segments, the parameter set was selected to cover the cases when the period of the wave is between 0.1 and 10 times the size of the tile size. There is no point going below 0.1 as the surface is practically a plane with gradient of 2π/T. The offset was studied to be between $10^{-10}$ and $10^{-1}$. The zero offset was not used, as in the $p_{i}=0$ case (Equation (1)) is not computable by machine; of course, its limit can be derived by l’Hospital’s rule, however for running time reasons the conditional branching for calculating the $p_{i}=0$ entropies was not implemented. The parameter set for φ was from 0° to 180°, and the $T_{y}/T_{x}$ ratio from 0.1 to 10.

As the bowel is a tube, and in perspective the waves of the wall might seem to be concentric, the circular, or elliptic waves are also of interest. The distribution for these waves is modeled as

(14) $$f_{C}\left(x,y\right)=\sin\left(\sqrt{{\left(\frac{2\pi }{T_{x}}\left(x-x_{0}\right)\right)}^{2}+{\left(\frac{2\pi }{T_{y}}\left(y-y_{0}\right)\right)}^{2}}+\varphi \right)+0.5+B.$$

**Figure 6:**
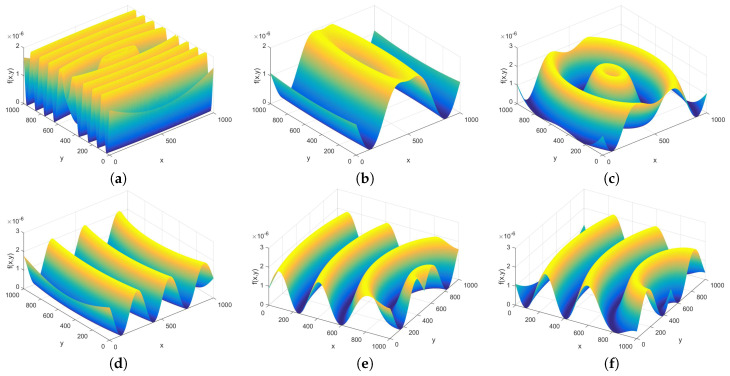
Picture of elliptical waves with minimum and maximum wavelength ratio in (**a**,**b**), minimum and maximum off-center positions in (**c**,**d**), and minimum and maximum phases in (**e**,**f**).

The six parameters are the two wavelengths $T_{x}$ and $T_{y}$, the two coordinates of the center $x_{0}$ and $y_{0}$, the phase φ and the offset *B*. The studied parameter domains for the ratio $T_{x}/T_{y}$ are from 1/5 to 5, for the center coordinates $\left(x_{0},y_{0}\right)$ from the center of the tile, i.e., from (0,0) to (N,0), and for the phase φ from 0° to 180°, as can be seen in Figure 6.

#### Tilted Waves

As in the pictures the further parts of the bowel are darker, waves with a tilt were also studied. In this case, instead of the constant offset *B*, a plane with a slight slope was also applied. The direction of the slope was perpendicular to the wave front, as mostly the wave fronts are perpendicular to the bowel axis. Plane tilt was given to the waves with straight front and conical tilt to the circular fronts. The parameter was the ratio of the wavelength and the gradient, which was between $2^{0}$ and $2^{-6}$.

### 3.3. Model Structures for the Polyps and their Shadows

**Figure 7:**
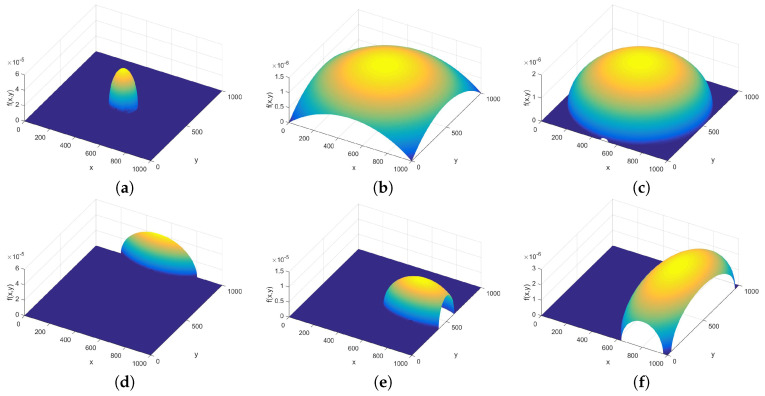
Picture of hemispheres. In (**a**,**b**) the radius is at the two extremum of the parameter set, in (**c**,**d**) the offset, and in (**e**,**f**) the deformation toward an ellipsoid.

The polyp can be quite well modeled as hemispheres, ellipsoid or sphere segments. The studied distribution was

(15) $$f_{S}\left(x,y\right)=\max\left(\sqrt{R^{2}-\left({\left(\frac{\left(x-r_{x}\right)}{R_{x}}\right)}^{2}+{\left(\frac{\left(y-r_{y}\right)}{R_{y}}\right)}^{2}\right)},B\right),$$

with *R* being the radius of a sphere, $R_{x}$ and $R_{y}$ the parameters distorting the hemisphere to a half ellipsoid, $\left(r_{x},r_{y}\right)$ the coordinates of the center of the object, and *B* the background height around the ellipsoid or sphere segment, which was usually set to $10^{-10}$. The analysis went on in two directions: first the size and the position of a hemisphere was varied, and then the positions remained at the center and at the edge of the picture, but the shape was distorted to ellipsoid. The the distributions corresponding to the limits of the parameter sets are given in Figure 7.

#### Model Structures for the Shadows around the Polyps

**Figure 8:**
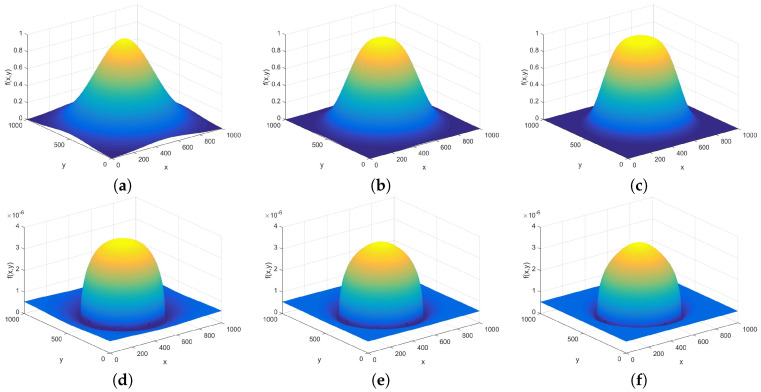
Picture of a Gaussian function (i.e., α=2 in Equation (16), (**a**)) together with its superposition with a hemisphere (**d**), as well as the α=3 (**b**) and α=4 (**c**), with their superpositions with hemispheres (**e**), and (**f**). The hemisphere has the same radius as the σ of the exponential functions, and the amplitude ratio between the hemisphere and the exponential is 3 to 1.

Only Gaussian functions were used for generating the valley representing the shadow around the spheres; however, distributions of type

(16) $$f_{G}\left(x,y\right)=\exp\left(-{\sqrt{\left(\frac{{\left(x-r_{x}\right)}^{2}}{\sigma_{x}^{2}}+\frac{{\left(y-r_{y}\right)}^{2}}{\sigma_{y}^{2}}\right)}}^{\;\alpha }\right),$$

were also studied. Here, the center was always set as the same position as the center of the half ellipsoid, and the variances $\sigma_{x}$ and $\sigma_{y}$ as the same as the radial parameters of the ellipsoid. As functions that use higher power α in Equation (16) have wider and flatter central part and quicker decrease, they were also tested for reproducing the shadows around the hemispheres, with less distortion in the spheres. However promising this idea was, the results were usually less similar to the real polyps than the α=2 case, as can be seen in Figure 8.

## 4. Discussion of the Structural Entropy Characteristic Curves

### 4.1. Characteristic Lines from Artificial Model Systems

After deciding the possible models and their parameter sets, the structural entropy versus spatial filling factor plots were studied. Two parameters were changed in one plot series: the first given as the third axis of the plot, and the second as the color and marker of the plotted points. Even though the parameter sets consist of discrete values, the points corresponding to the second parameter value and varying along the first parameter were connected as a guide of the eye. Most of the characteristic curves are presented in Appendix A and Appendix B. The reason for this is manifold. First, with only the title containing the information about the model type, it is easier to see the result. A similar statement is true for the text about the characteristic curves in this section: not breaking the text with images helps keep the focus. Second, there are many parameter combinations that do not seem to be very important at this point, and their results can be summarized in one sentence. Third, usually three or four plots are given for one result, which is too many; however, the 3D plots with parameter–filling factor–structural entropy axes are usually interesting not only from one point of view, but from the three projections and one perspective plot as well.

### 4.2. Dependence on the Image or Tile Size

The dependence on the tile size can be excluded as a parameter if all the other parameters are given in the relation to the tile size *N*. The only exception is the offset parameter *B*. In the cases when *B* was used only for technical reasons, namely for treating the $p_{i}=0$ cases without having to use if–then conditional branchings in the program, the offset should be small enough to be negligible compared to the rest of the intensity values. To set a suitable default offset, the tile size dependence of the structural entropy and the spatial filling factor of all our model surfaces were determined. In all cases, we found that, between the realistic limits of N=20 (smallest applied tile size in the case of lower resolution images in [33]) and N=1000 (magnitude of the full image size of database [24]), the tile size–lnq–$S_{str}$ curves are practically the same if *B* is smaller than $10^{-5}$.

**Figure 9:**
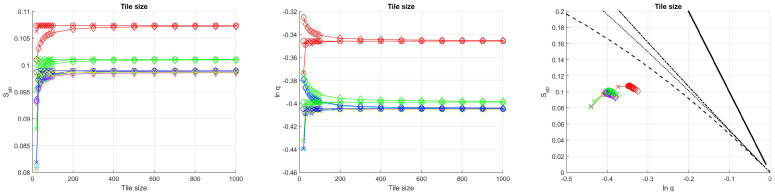
The structural entropy $S_{str}$ and the logarithm of the spatial filling factor *q* versus the tile size for straight waves. The offsets are the following: red, 0.1; green, 0.01; blue, 0.001; cyan, 0.0001; magenta, 0.00001; yellow, 0.000001. The last three are barely distinguishable. The wavelength to tile size ratios are the following: ⋄: 2, ∘: 1, ×: 0.5.

An example (of waves with straight wavefront) is plotted in Figure 9. This serves as a demonstration of how the three projections of the 3D plot look similar. It can also be seen in Figure 9 that, although above the tile size N=200 the values lnq and $S_{str}$ are almost completely independent of the tile size (even of the tile size to wavelength ratio), in the region of smaller *N*, the tile size plays not negligible role, thus we can conclude that using larger tile sizes in the evaluation process makes the results more stable and reliable. However, fulfilling the condition of using at least 200 by 200 pixel sized tiles is not always possible, especially if the images are of 384×288 size, such as in the case of the CVC Clinic database [27].

### 4.3. Dependence on the Wavelength Compared to the Image Size

If the wavelength *T* of the wave in the picture segment is chosen as the first parameter, it can be either larger than the tile size *N*, or at most one third of it, thus the tile size to wavelength ratio was selected to be between 1/4 and 3. In the case of *N* to *T* being 1:4, the resulting wave distribution starts to resemble a plane; this was the reason the parameter space was extended to 1:10 limit, as in that case the limiting behavior could also be studied. In addition, the other limit was extended compared to the realistic case to see whether there is a limiting behavior in the small wavelength domain as well.

**Figure A1:**
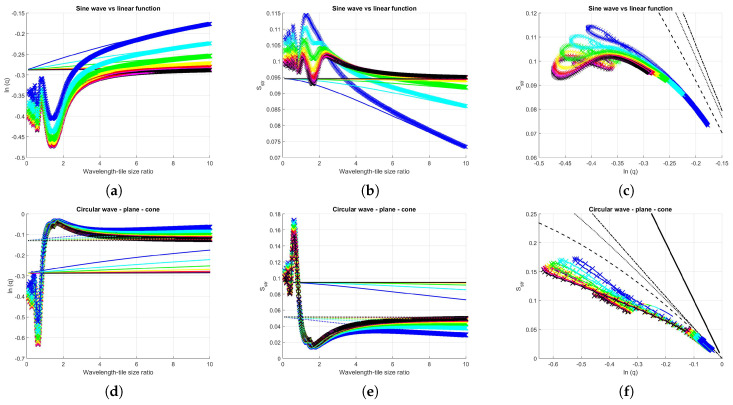
Characteristic curves of waves with straight (**a**–**c**) and circular (**d**–**f**) wavefronts, if the parameters are the wavelength to tile size ratio and the constant offset. The colors denote the following constant offsets: B=0.1, blue; B=0.05, cyan; B=0.025, green; B=0.0125, yellow; B=0.00625, red; B=0.003125, magenta; B=0.0015625, black. As a reference, the characteristic curves for planes with the same gradient as the x→0 gradient of the waves are plotted with solid lines without markers, whereas the cones corresponding to the initial slope of the circular waves are plotted in dotted lines.

**Figure A2:**
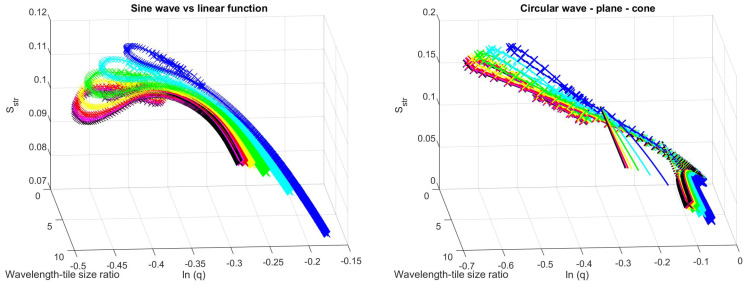
3D view of Figure A1.

The offsets can also play important role if they are larger than 0.001, thus, as a first step, the second parameter was selected to be *B*. The characteristic curves for both the waves and the limiting planes are given in Figure A1 and Figure A2 in the Appendix, both for straight and for circular wavefronts. Figure A1 gives the three projections of the $S_{str}\left(\ln q,T/N\right)$ plots, while Figure A2 shows 3D perspective. It can be seen that in the large T:N domain the curves follow their corresponding limits’ characteristics (i.e., the planes for the straight wave front and the cones for the circular wavefront). The small T:N ratio part of the curve oscillates around a value with decreasing amplitude in both lnq and $S_{str}$, resulting in ribbon-bow-like, eight-shaped loops in the $S_{str}\left(\ln q\right)$ plots.

**Figure A3:**
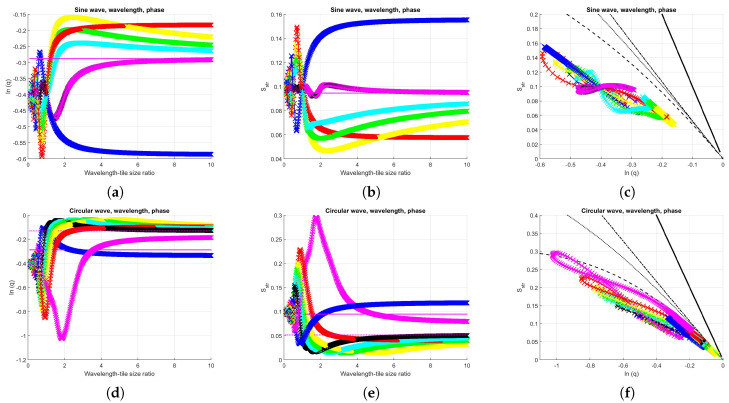
Characteristic curves of waves with straight (**a**–**c**) and circular (**d**–**f**) wavefronts, if the parameters are the wavelength to tile size ratio and the phase φ. The colors denote the following: $\varphi =0^{\circ }$, black; $\varphi =30^{\circ }$, cyan; $\varphi =45^{\circ }$, green; $\varphi =60^{\circ }$, yellow; $\varphi =90^{\circ }$, red; $\varphi =180^{\circ }$, magenta; $\varphi =270^{\circ }$, blue. As a reference, the characteristic curves for planes with the same gradient as the x→0 gradient of the waves are plotted with solid lines without markers, whereas the cones corresponding to the initial slope of the circular waves are plotted in dotted lines.

**Figure A4:**
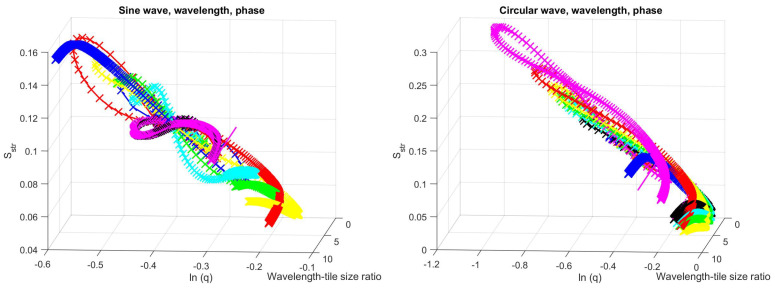
3D view of Figure A3.

The second parameter can also be the phase. With a very small offset $B=10^{-10}$; the characteristic curves can be seen in Figure A3 and Figure A4. It is clearly visible that phase influences the structural entropy, and spatial filling factor values, especially in the lower T:N domains.

**Figure A5:**
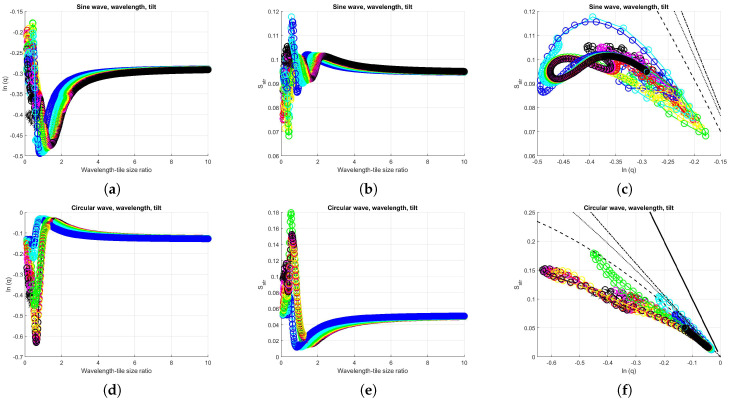
Characteristic curves of waves with straight (**a**–**c**) and circular (**d**–**f**) wavefronts, if the parameter is the wavelength to tile size ratio and the tilt gradient. The colors denote the following linear offset slopes to wavelength ratios: B/T=1, blue; B/T=0.5, cyan; B/T=0.25, green; B/T=0.125, yellow; B/T=0.0625, red; B/T=0.03125, magenta; B/T=0.015625, black. As a reference, the characteristic curves waves without tilt are plotted in thinner lines with different × markers.

**Figure A6:**
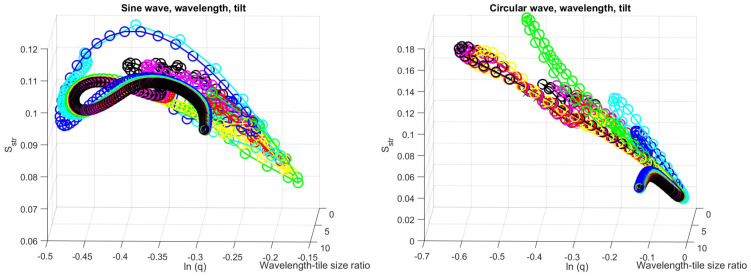
3D view of Figure A5.

Another possibility for the other parameter beside the wavelength is the tilt slope to wavelength ratio. The results are summarized in Figure A5 and Figure A6 in the Appendix. It can be seen that, for wavelengths larger than the tile size, the tilt does not have real influence, however, in the small wavelength direction, the characteristic curve oscillates much more vehemently if tilt is present than in the tiltless cases, and also some points with very high curvature—turning points—arise in the case of the waves with tilt.

### 4.4. Dependence on the Phase and Center-Shift

In the case of the colonoscopy image categorization, besides dividing the pictures into fixed tiles, applying sliding tiles and analyzing the characteristics of the arising $S_{str}\left(q\right)$ or $S_{str}\left(\ln q\right)$ point set is another option. This can be represented as a phase scanning in the case of the waves with straight wavefront, and as moving the center in the case of the circular waves.

**Figure A7:**
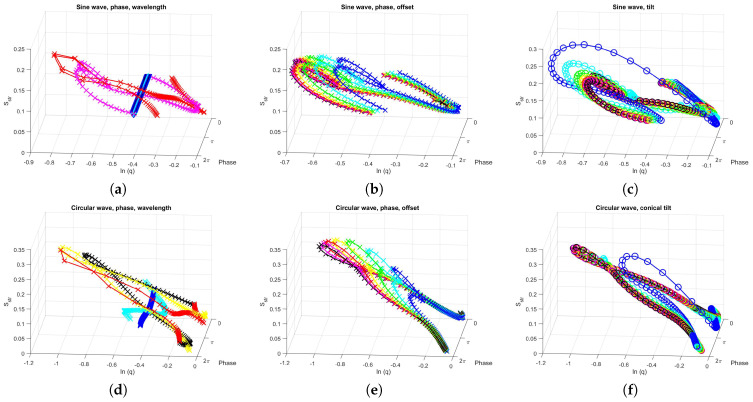
Characteristic curves of waves with straight (**a**–**c**) and circular (**d**–**f**) wavefronts, if the parameter is phase. The other parameters are the following. (**a**,**d**): wavelength. The colors denote the following wavelength to tile size ratio: T/N=0.1, blue; T/N=0.5, cyan; T/N=1, black; T/N=2, yellow; T/N=10, red. (**b**,**e**): constant offset. The colors mean the following: B=0.1, blue; B=0.05, cyan; B=0.025, green; B=0.0125, yellow; B=0.00625, red; B=0.003125, magenta; B=0.0015625, black. (**c**,**f**): linear offset. The colors mean the following: B/T=1, blue; B/T=0.5, cyan; B/T=0.25, green; B/T=0.125, yellow; B/T=0.0625, red; B/T=0.03125, magenta; B/T=0.015625, black. The markers × denote the zero-tilt limit, while the circles show the results for the tilted waves.

For the studies of the phase, the fourth dimension can be either the wavelength, or the offset (constant or linear). For all three cases (i.e., wavelength, offset, and tilt), only the 3D plots are given in Figure A7 of Appendix A. The non-varying parameters were set the following way. The offset in the first image, where the wavelength varied, was set to be negligible ($10^{-10}$). The wavelength in the second column, where the offset varied, was set to 2N, as for wavelength values smaller than *N* neither the structural entropy nor the spatial filling factor had dependence on wavelength, in the case of the straight wavefront, and very simple sinusoid wave-like characteristic curves arise in the case of the circular wavefronts. In both cases, if the wavelength is larger than the tile size, the loops formed on the $S_{str}\left(\ln q\right)$ plot are turned back at a point, resulting in hook-like lines, which seem to have derivative singularities, or at least very rapid variation in their gradients. As can be seen in the plot with varying offset, if the offset becomes negligible, this turning point becomes a simple inflection on the characteristic line. In the case of the linear offset (tilt) of the third column, the hook-like behavior becomes rather loop-like.

However, as both the offset and the tilt can easily be removed from a picture by image processing means (the offset by a counter-offset, i.e., by setting the minimum of the pixel intensities as 0, whereas the tilt by removing a mean-filtered version of sufficiently large filter size from the image), it is more advisable to remove these unnecessary information sources from the picture.

**Figure A8:**
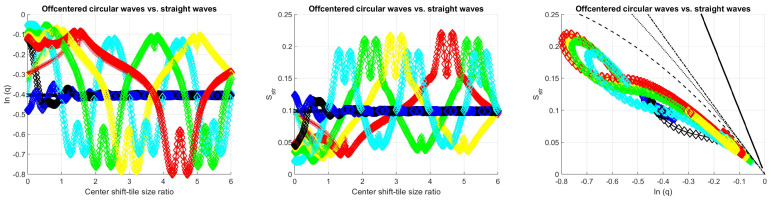
Characteristic curves of circular wavefronts, if the parameter is center shift $x_{0}$ and wavelength to tile size ratio. In the first row, the other coordinate of the center shift was set to $y_{0}=0$ and the colors denote the following wavelength to tile size ratios: T/N=0.5, blue; T/N=1, black; T/N=2, cyan; T/N=3, green; T/N=4, yellow; T/N=6, red. The markers are ⋄. As a reference, the characteristic curves of straight waves of same parameters were plotted with thinner lines and smaller markers of shape × (the phase had to be set to π to match the curves). As a reference, the characteristic curve of a straight wave with the same parameters was plotted with thinner black line and smaller markers of shape × (the phase was again π).

**Figure A9:**
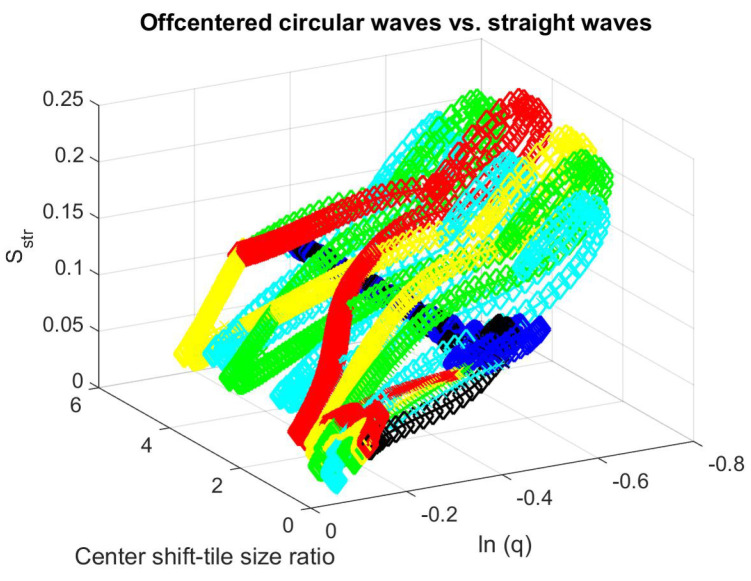
3D view of Figure A8.

Moving along a diameter of a circular wave results in the characteristic lines given in Figure A8 and Figure A9. Both the lnq and the $S_{str}$ curves are periodic at the higher center shift domains, and, similar to the straight waves, they have hook-like characteristics, if the wavelength is larger than the tile size. In the case of smaller wavelengths, the oscillations are of much smaller magnitude.

**Figure A10:**
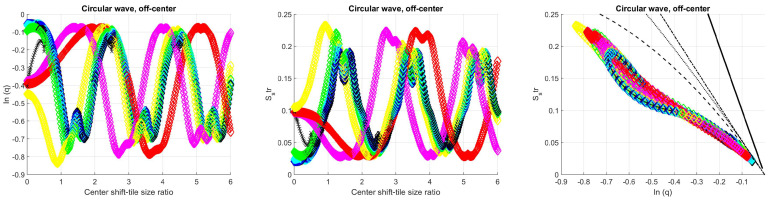
Characteristic curves of circular wavefronts, if the parameter is center shift $x_{0}$ and center shift in the other direction. The wavelength to tile size ratio was set to T/N=2, and the colors mean the following other coordinate of the center shift to tile size ratios, $y_{0}=0$ and the colors denote the following: $y_{0}/N=0$, black; $y_{0}/N=1/8$, blue; $y_{0}/N=1/4$, cyan; $y_{0}/N=1/2$, green; $y_{0}/N=1$, yellow; $y_{0}/N=2$, magenta; $y_{0}/N=4$, red. The markers are ⋄. As a reference, the characteristic curve of a straight wave with the same parameters was plotted with thinner black line and smaller markers of shape × (the phase was again π).

**Figure A11:**
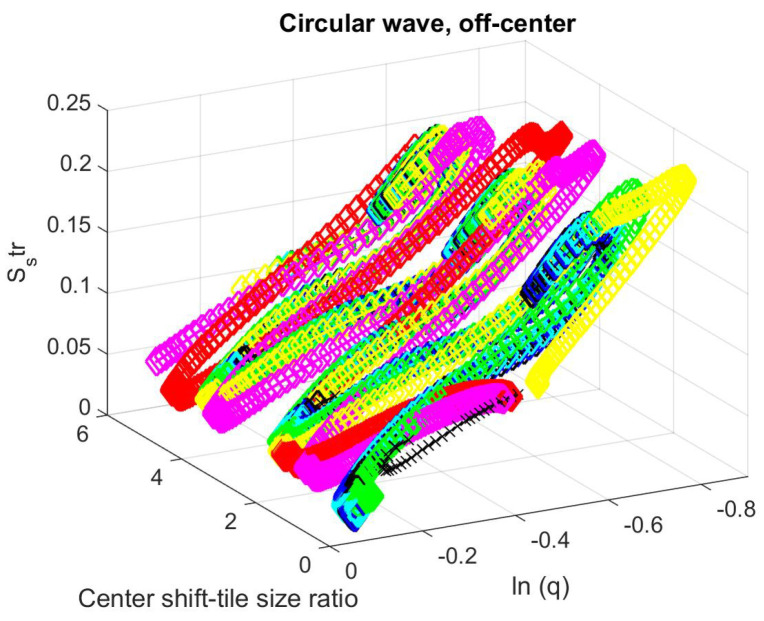
3D view of Figure A10.

If the center is moved in the other direction as well, the upper hook becomes more and more asymmetric, and a shift also appears, as is demonstrated in Figure A10 and Figure A11 of Appendix A.

As in the case of scanning a row of a picture the tile size is usually smaller than the wavelength, the large wavelength curves are of greater interest from the point of view of polyp detection. In addition, as the center of the elliptical waves are generally in the more distant parts of the image, i.e., practically never in the same frame as the polyp, the offsets larger than the tile size are of more interest. In these cases, as can be seen in Figure A10 and Figure A11, the straight waves model the behavior of the circular waves very well.

**Figure A12:**
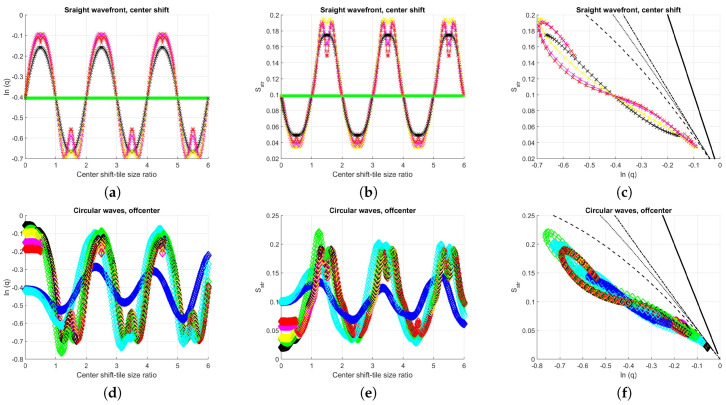
Characteristic curves of straight (**a**–**c**) and circular (**d**–**f**) wavefronts, if the parameters are the center shift and the direction of the wavefront. The colors denote the following ratios: $T_{y}/T_{x}=1/8$, blue; $T_{y}/T_{x}=1/4$, cyan; $T_{y}/T_{x}=1/2$, green; $T_{y}/T_{x}=1$, black; $T_{y}/T_{x}=2$, yellow; $T_{y}/T_{x}=4$, magenta; $T_{y}/T_{x}=8$, red.

**Figure A13:**
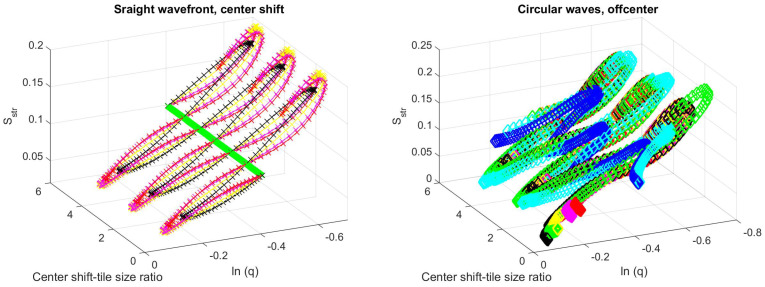
3D view of Figure A12.

The dependence directions of the straight waves and the axis ratio of the elliptic waves can be seen in Figure A12, and in its 3D version in Figure A13. We can conclude that the direction does not influence the characteristic curves of straight waves if the wavelength is below the tile size. The hook-like characteristic curves with smaller or larger asymmetry remain for both the straight and the circular waves, and for the elliptical waves the ratio of the axes becomes negligible if the center is shifted out of the tile.

To summarize this subsection, scanning the picture with a moderately large window along a line or column can be of greater interest from structure detection point of view. In this case, for larger distance of the center of the elliptical waves, they behave similarly to the waves with straight wavefront: periodic, hook-like characteristic curves are usual, which an be distorted by other parameters.

### 4.5. Hemispheres

**Figure A14:**
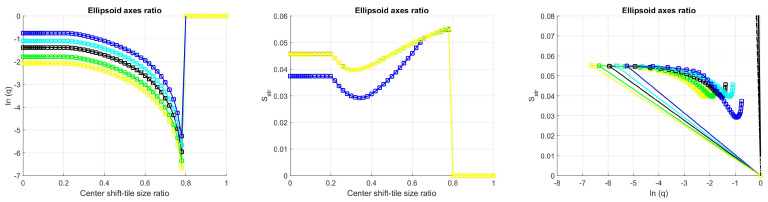
Characteristic curves of hemispheres, if the parameter is center shift $x_{0}$. The other coordinate of the center shift was set to $y_{0}=0$. The colors denote the following axes ratios: $R_{y}/R_{x}=0.5$, blue; $R_{y}/R_{x}=0.75$, cyan; $R_{y}/R_{x}=1$, black; $R_{y}/R_{x}=1.5$, green; $R_{y}/R_{x}=2$, yellow. *R* was set to 0.3N, while $R_{y}$ was set to 1.

**Figure A15:**
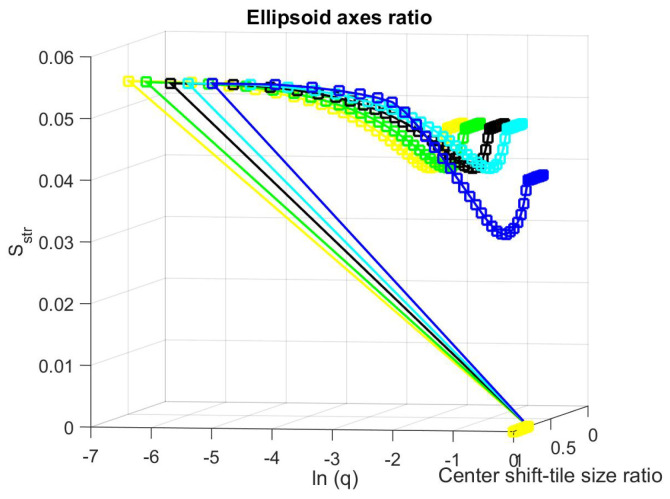
3D view of Figure A14.

In the case of the sphere or ellipsoid segments, the parameters we selected are the radius to tile size relation, the ratio of the axes of the ellipsoid and the center shift. The characteristic curves can be seen in Figure A14 and Figure A15 of Appendix B. It can be seen that the hemispheres or half ellipsoids have very low structural entropy because, in the case of a sphere with radius to tile size ratio 0.3, a very large part of the picture is completely flat and dark, with 0 entropy (and thus 0 structural entropy). In addition, the radius and axis ratios influence only the spatial filling factor; the structural entropy does not change as long as the whole ellipsoid is within the tile.

**Figure A16:**
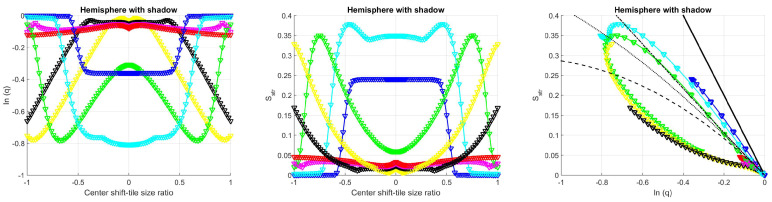
Characteristic curves of hemispheres with Gaussian shadows, if the parameter is center shift $x_{0}$ and radius to tile size ratio. The other coordinate of the offset was set to $y_{0}=0$. The colors denote the following radius to tile size ratios: R/N=0.1, blue; R/N=0.25, cyan; R/N=0.5, green; R/N=0.75, yellow; R/N=1, black; R/N=1.5, magenta; R/N=2, red.

**Figure A17:**
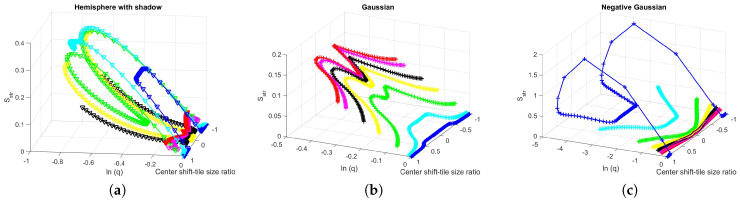
(**a**) 3D view of Figure A16; (**b**) Gaussian functions; and (**c**) negative Gaussian functions. The variances of the Gaussians are the same as the square of radii of the hemispheres. The color notation is the same as in Figure A16. The ratio of the Gaussian’s height and the radius is 1:3.

If the shadow part of the picture is also included into the model, i.e., the Gaussian like functions (Equation (16)) are subtracted from the hemispheres, the structural entropy of course becomes much larger, as the part of the image with zero pixel intensity becomes very small. The results are given in Figure A16 and Figure A17 of Appendix B. The characteristic curves of the Gaussian-like structures and their negative counterpart are also given, but only in the 3D plot form.

The Gaussian distributions are on their theoretical characteristic line for that central region, where the $S_{str}\left(\ln q\right)$ points are constant, and deviate from their theoretical value if significant part of the distribution is outside of the tile (the deviation starts to be visible at the shift of about 5R and in very small variation cases, if the shift is larger than about eight times the radius, the structural entropy’s deviation starts to grow, and then its value sinks to the origin of the plot.

The structural entropy plots of the hemispheres with shadow have big loops if their radius is small, and hooks start to form with the increasing of the size of the polyp model. The Gaussian-like structures with higher power α were also tested, however, their result did not differ much from the Gaussian case, only the loop area became a little bit smaller, as the power increased.

**Figure A18:**
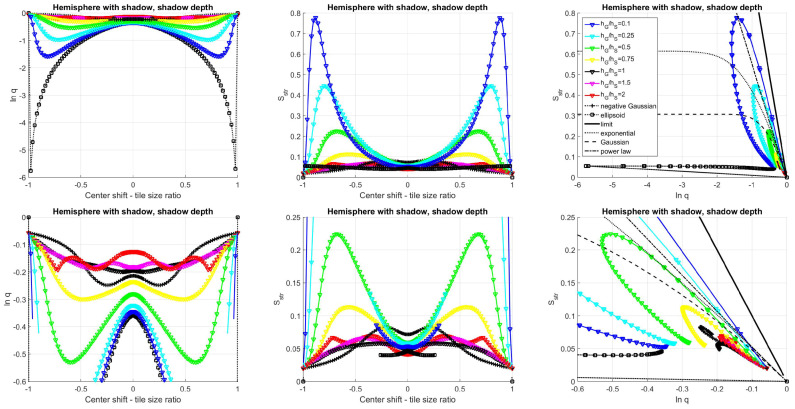
Characteristic curves of hemispheres with Gaussian shadows, if the parameters are center shift $x_{0}$ and ellipsoid to Gaussian height ratio $h_{G}/h_{S}$. The lower row is the zoomed version of the upper one. All pictures use the same markers with the same meaning as given in the upper right hand side subplot.

**Figure A19:**
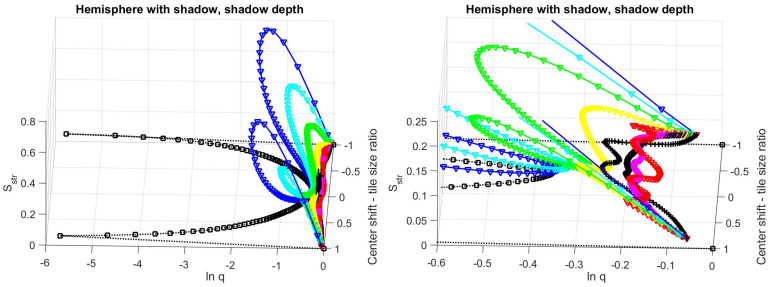
3D version of Figure A18 both for the full and the zoomed image.

In addition, the depth of the shadow, or the shadow to ellipsoid height ratio, is interesting. If the shadow is much deeper than the polyp, we arrive at the distant part of the bowel, the tunnel, which almost always has a darkening part and a turning, which often appears as hemisphere or similar object in the cross section of the distribution. The results are plotted in the Appendix in Figure A18 and Figure A19. The pictures show that, as the shadow deepens, the hooks at the sides of the hemisphere decrease, moving inward, toward the point with 0 center shift. In addition, more smaller hooks appear in the inner domain.

**Figure A20:**
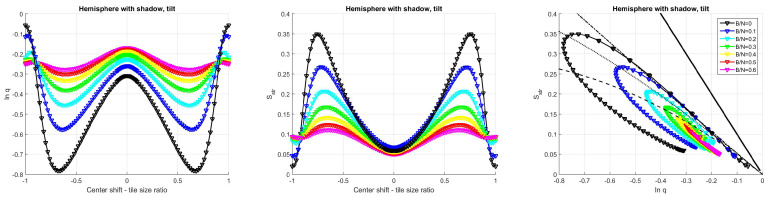
Characteristic curves of hemispheres with Gaussian shadows, if the parameters are center shift $x_{0}$ and tilt gradient *B*.

**Figure A21:**
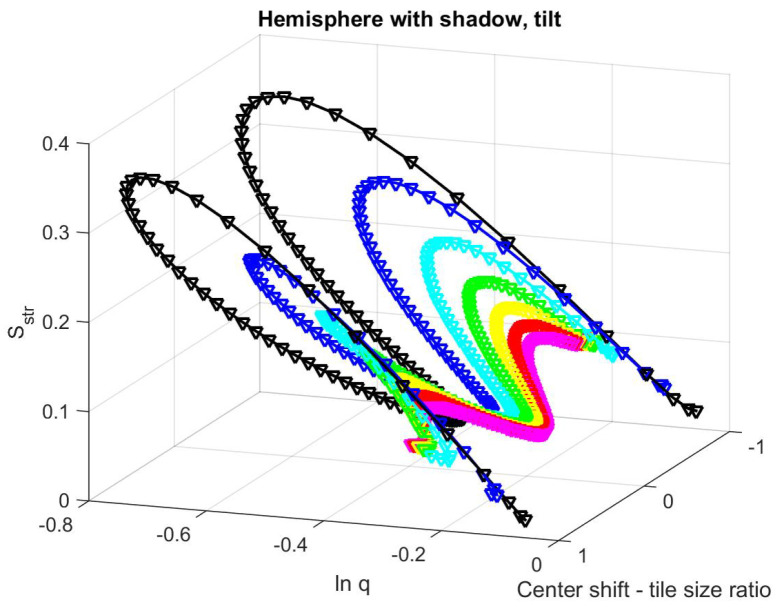
3D version of Figure A20.

Tilt is important in this case, too. The effects of introducing and increasing tilt are shown in Figure A20 and Figure A21 of Appendix B. It is clearly visible that the distance of the received $S_{str}\left(\ln q\right)$ points decrease from the origin, and other little hooks emerge in the middle region of the plot. This is of course not always this visible: if the radius is too small, the hooks disappear here as well, such as in the case of the blue curve in Figure A16. This means that, if the polyp is much smaller than the window used for scanning, it behaves completely differently from the ones with radii more similar to the tile size.

**Figure A22:**
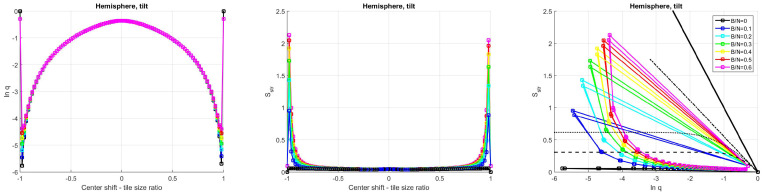
Characteristic curves of hemispheres, if the parameters are center shift $x_{0}$ and tilt gradient *B*.

**Figure A23:**
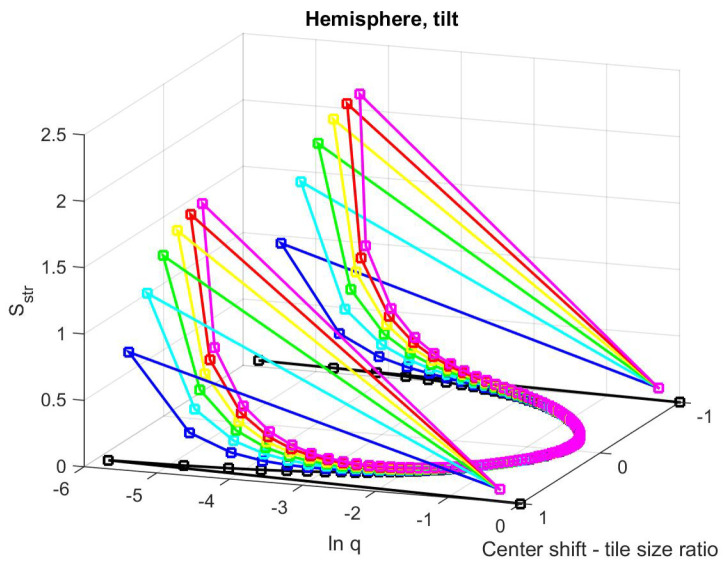
3D version of Figure A22.

**Figure A24:**
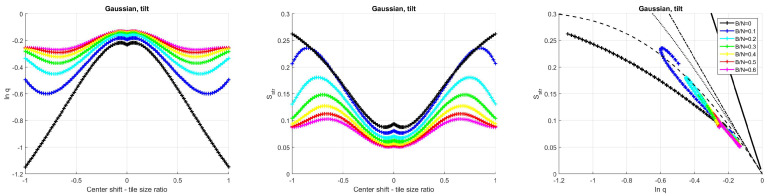
Characteristic curves of Gaussian distribution segments, if the parameters are center shift $x_{0}$ and tilt gradient *B*.

**Figure A25:**
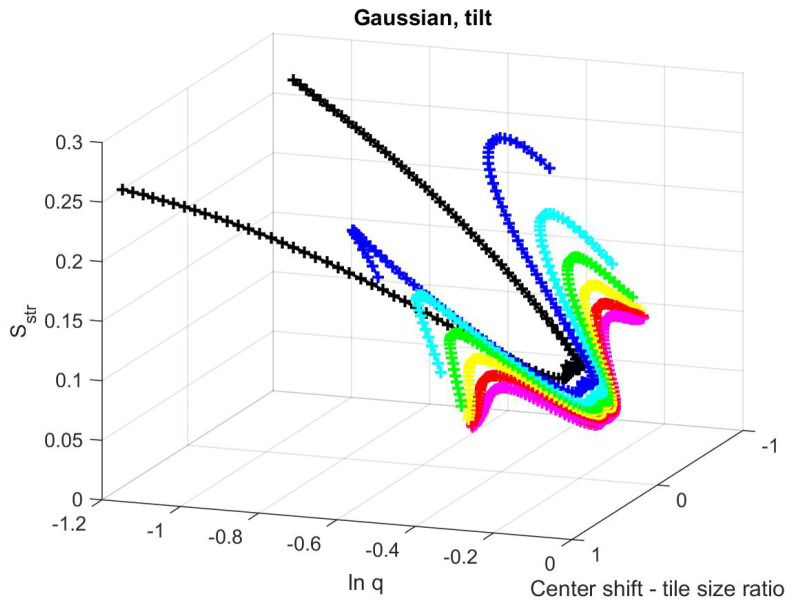
3D version of Figure A24.

**Figure A26:**
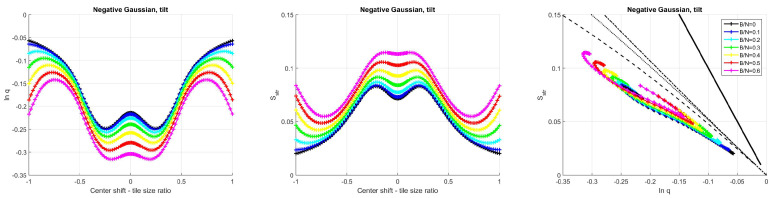
Characteristic curves of the negative Gaussian shadows, if the parameters are center shift $x_{0}$ and tilt gradient *B*.

**Figure A27:**
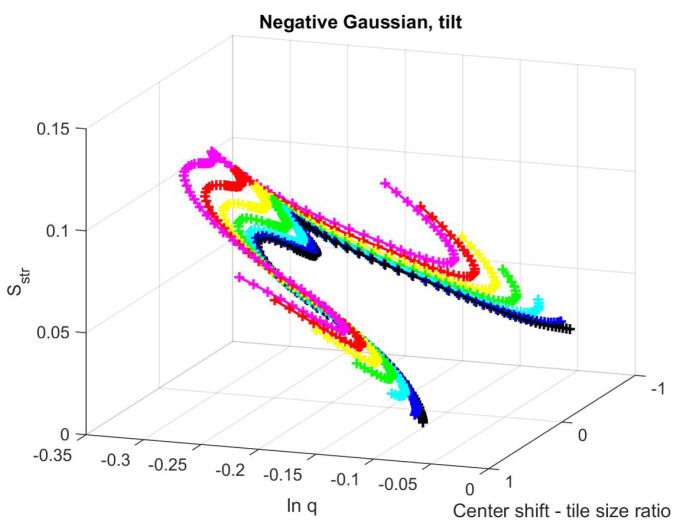
3D version of Figure A26.

The components of the hemisphere with Gaussian shadow were also studied and the effect of the tilt to their properties are given in Figure A22, Figure A23, Figure A24, Figure A25, Figure A26 and Figure A27. In the case of the hemispheres, the tilt increases the structural entropy and decreases the spatial filling factor, thus elevating the hemisphere’s curve from the lnq axis. In the case of the Gaussians, the magnitude decreases with increasing tilt. In those center shift values, where the sphere dominates in the tile (i.e., when the tile center is around the center of the sphere), the movement toward the origin is less than those parts that contain picture domains with 0 value.

### 4.6. Superpositions

The superpositions of the semi-ellipsoids and the waves have rather complex behavior, depending on which component is dominant according to the magnitude and size. If the wavelength is larger than the tile size, and the hemisphere diameter is smaller, then the setup is very similar to a roundish polyp largely protruding into the bowel volume. If the wavelength is smaller, the arising picture is similar to those flatter polyps, which are sitting at the bends of the bowel wall, making these bends only slightly thicker at a given region.

**Figure A28:**
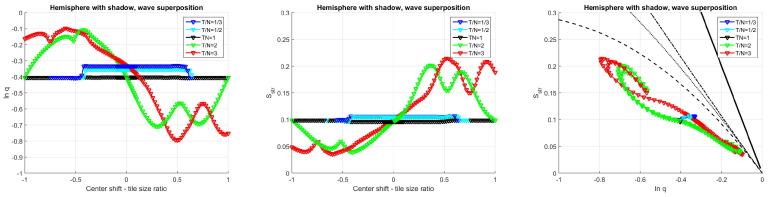
Hemisphere with gaussian shadow superposed to a wave of wavelength *T* with picture segment size N×N. The radius was set to 0.1N. The small wavelength curves are similar to polyps sitting on a bending of the bowel wall making it slightly thicker. The large wavelength lines are similar to roundish polyps sitting on slightly curved bowel walls.

**Figure A29:**
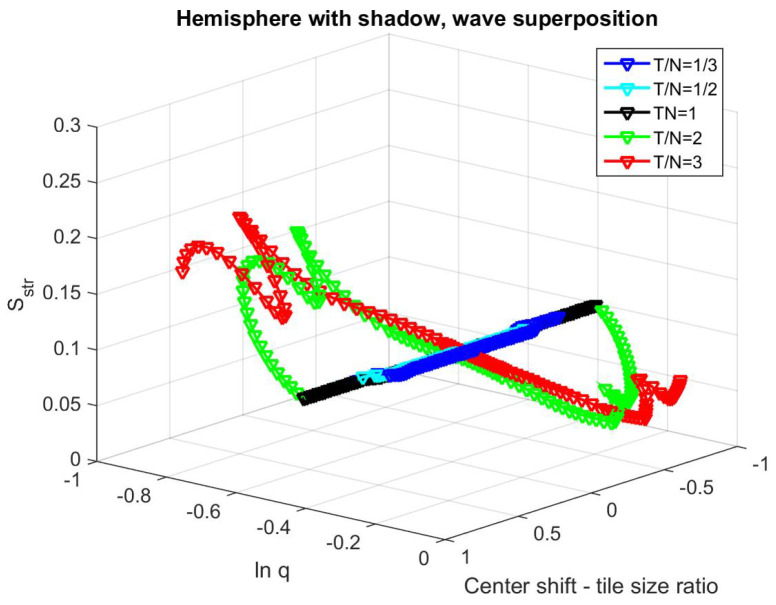
3D version of Figure A28.

Characteristic curves for such superstructures can be seen in Figure A28 and Figure A29 of Appendix B.

The resulting characteristic curves are also of two types: for the larger wavelengths, the periodic behavior dominates, hooks similar to the ones in Figure A8 appear, and the sphere segment and its shadow causes only slight asymmetries. If the wavelength is smaller that the tile, the two components can decrease each other’s structural entropy and filling factor.

The Rényi entropy based structural entropy and the spatial filling factor is able to distinguish parts of a superstructure, if they are multiplied and not added. In the case of a multiplicative superstructure, the $S_{str}$ and lnq values of the components are simply added together. Unfortunately, in the case of additive superstructures, the $S_{str}$ and lnq values of the component structures can only be detected, if one of the structures is dominant. It might also be possible to detect components of the superstructures, if they are of different characteristic lengths, and wavelet analysis or other filtering method is used to separate the different characteristic lengths [59].

### 4.7. Summary of the Artificial Surface Characteristic Curve Properties

For a better visibility, we summarize the previous results in Table 1, concentrating on how other parameters influence the center-shift curves.

### 4.8. Typical Characteristic Curves of Real Images

**Figure 10:**
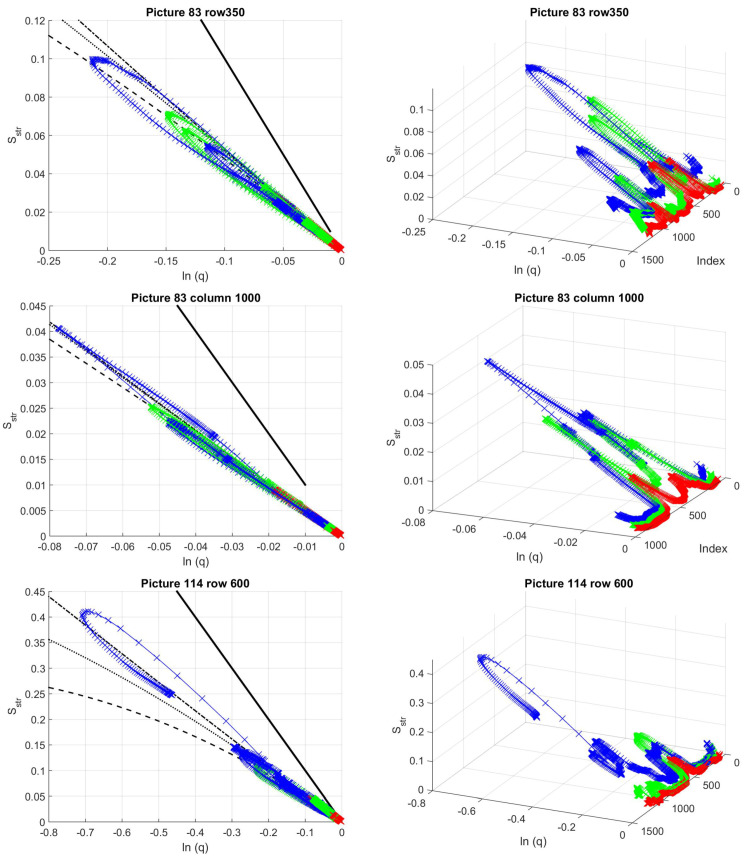
Structural entropy across real polyps. The center of the sliding tile is at the curves plotted in Figure 3. Fixed tile size of 50 by 50 pixels. The polyps are located approximately between pixel indices 900 and 1100 for picture 83, row 350, between 300 and 500 for the same picture’s 1000th column, and between 600 and 1100 for picture 114, row 600.

In the case of real images, instead of fixed tiles, we applied the sliding tile method suggested in Section 4.4. In Figure 10, the two cuts in Figure 3 are scanned with tiles of size 50 by 50. The characteristic curves of these cuts are very similar to the ones given in Figure A16, however, as the distant, dark part of the bowel is also similar to the hemisphere with shadow model profile in some cases (see pixels 200-600 in picture 83, row 350), such occasions may cause misinterpretation of the $S_{str}\left(\ln q,i\right)$ curves, and thus false positive categorization.

**Figure 11:**
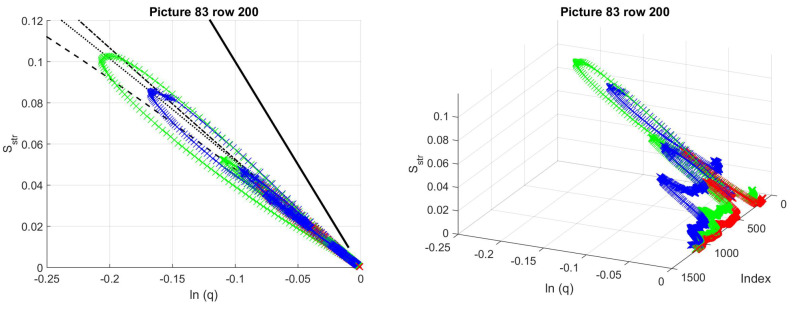

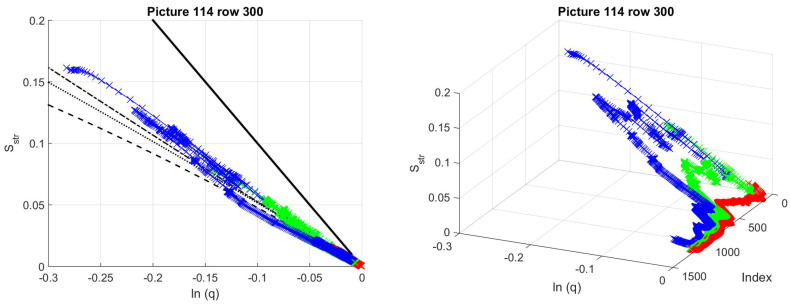
Structural entropy across pictures in scans without polyps. The center of the sliding tile is at the rows 200 and 300 of the pictures in Figure 2. Fixed tile size of 50 by 50 pixels. The dark part mimicking polyp is approximately located between pixel indices 300 and 700 in picture 83, row 200, while the waves are between pixels 200 and 800 in picture 114, row 300.

In Figure 11, two scans without polyp are given as an example, one with clearly distinguishable waves, and the other across a polyp-like appearing curvature of the bowel, where the shadows are much larger, and the spherical characteristics are much weaker than in real polyps. These polyp mimicking parts with much more emphasized shadows generally have larger loops than the real polyps. The waves do not have such expressed, curvy hook-like behavior as can be seen in Figure A10 and Figure A11.

### 4.9. Real Picture versus Model: The Applicability

As an example, part of a real picture was studied. As we suggest removing the offset and the tilts from the image by shifting the 0 level and applying a larger scale mean filtered version of the image, first we show their effects on the structural entropy–filling factor plots. We used the same 1000th column of image 83 from database [24], as in the previous section. The tile size remained 50 by 50.

**Figure 12:**
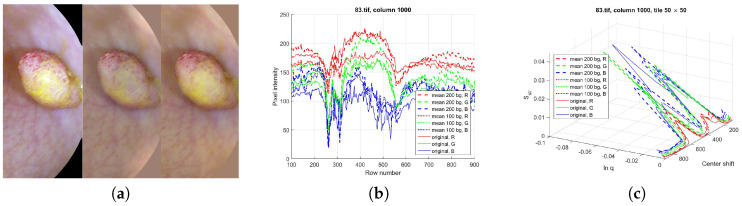
(**a**) A cut from the not processed, and background subtracted versions of picture 83.tif from database of [24]. The second picture segment’s background is generated by a 100 × 100 sized mean filter, the third slices by a 200 × 200 sized one. (**b**) The cross sections at the studied row before and after background subtraction. (**c**) Structural entropy across the 1000th column of the original picture, approximately at the middle of the cuts. Tiles of 50 by 50 pixels.

The image preprocessing algorithm consists of only the following steps: reflection removing, histogram stretching and removing of the mean-filtered background pattern [60]. As the image size is around 1000 in both directions, we applied filter sizes of 100 by 100 and 200 by 200. The pictures, the cross section cuts and the structural entropy–filling factor–scanning window center position plots are given in Figure 12. The average diameter of the polyp is also around 200–250 pixels, thus the background generated with the smaller filter size suppressed the sphere-like characteristics of the polyp, as can be seen in Figure 12a,b. The symmetrizing effect of removing the tilt from the background can also be seen, even with such rough background subtracting algorithm.

As the image preprocessing method was applied to the whole picture, not only to the shown segment, there is still an offset in the color channels, about 150 in the red channel, 80 in the green, and 50 in the blue. We used a simplified model to demonstrate that the characteristic curves of model systems are similar to those of the real images, even though many aspects of the real picture, such as the fine patterns, the details of the background, or the yellowish spot that causes a depression in the middle of the polyp in the blue channel of the original picture, are neglected.

In the model system, a hemisphere was used with Gaussian, or higher-order exponential shadow, and flat, constant offset. The offset values were chosen according to the picture color channels’ offsets. The polyp diameter was selected to be 120 pixels, and the height of the hemisphere to be 80 for the red, 120 for the green and 100 for the red channel. The shadows were Gaussian in both the red and the green picture parts, and third-order exponential for the blue part. The depth of the shadows were adjusted to be 30, 60 and 80 for the R, G, and B channels, respectively.

**Figure 13:**
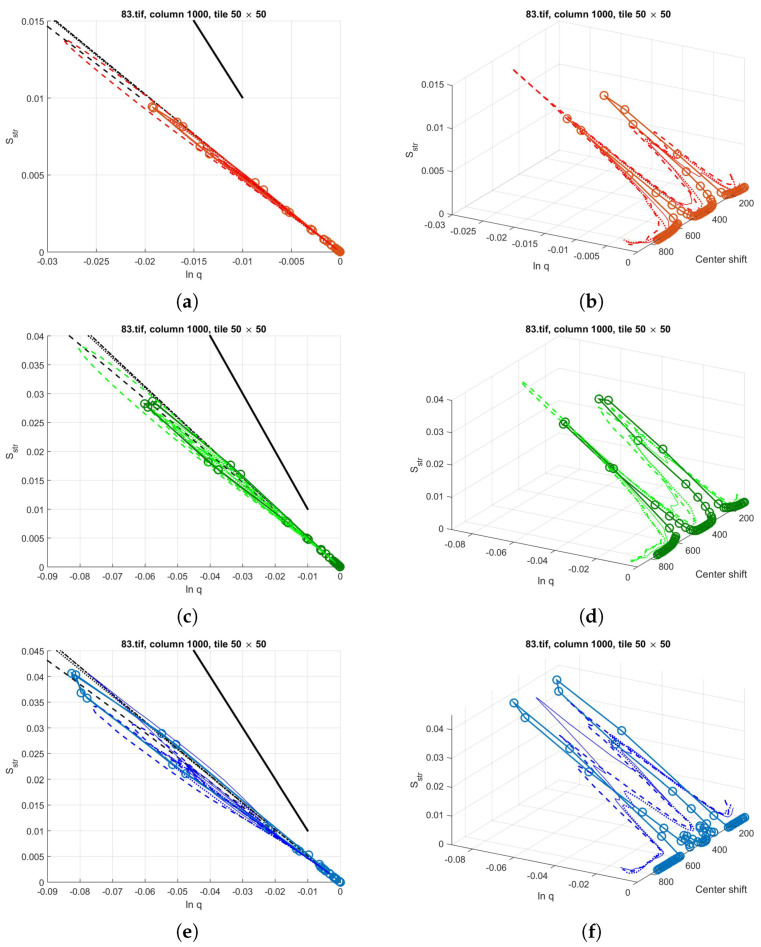
Rényi entropy based structural entropy–spatial filling factor–scanning window center curves for picture 83.tif from database of [24] in continuous lines. The preprocessed-image segments are given in Figure 12 in dashed and dotted lines. The structural entropy and filling factor values of the model system of hemisphere with Gaussian shadow and constant offset model systems. (**a**,**b**): red channel, (**c**,**d**): green channel, (**e**,**f**): blue channel. The shadows in the red and green channels are modeled with Gaussian functions of α=2, while for the blue channel, a higher, α=3rd order polynomial was used in the exponential function.

The resulting characteristic curves for the three color channels can be seen in Figure 13. From the $S_{s}tr\left(\ln q\right)$ plot, one might conclude that the models fit the real image very well, however, from the 3D curves, it can be seen that the fine structure of the structural entropy and filling factor around the shadow-polyp transition is not too well represented. As the fine-scale behavior of the pixel intensity distribution is not studied, these deviations may be attributed to the smaller sized patterns, however, this aspects needs further investigation. As the fine-scale pattern is useful in the case of pit pattern based classification, we decided not to study this problem in this article.

## 5. Conclusions

The shape of a probability distribution can be characterized by Rényi entropy based quantities, which are called spatial filling factor and structural entropy. Although the name is similar to the graph neighborhood relation based structural entropy that appeared earlier in the literature, these quantities of the same name are significantly different.

The Rényi entropy based structural entropy uses the native probability distribution of an image, i.e., its pixel intensity distribution, simply normalized in a manner that it would form a probability distribution. This structural entropy is from one point of view more complex than the graph theory based one, as it uses generalized, Rényi entropy differences instead of Shannon entropy. The main advantage of the method, however, is its simpleness. The probability distribution used in the entropies is straightforward, easy to generate, and does not need topological knowledge and neighborhood statistics, which might change with the resolution. These Rényi entropy differences can possibly be used as input parameters for fuzzy, support vector machine, or other metaheuristical or learning algorithms.

The application of the Rényi entropy based structure parameters requires plotting the structural entropy as the function of the filling factor, and comparing the result with existing characteristic curves. This process is easy to be visualized, but might be rather hard to understand and apply. Simply using the two quantities as input parameters for classification methods might loose a lot of information, which lies in the position on the $S_{str}\left(q\right)$ map related to characteristic lines. If this information is also to be included into the analysis, previous knowledge about the possible shapes are necessary to know which characteristic lines should be used as references, as these characteristic lines might overlap. This is the main disadvantage of the method.

Characteristic curves of simple distributions such as the Gaussian or exponentially decreasing probability distributions were known for a long time; however, distributions related to structures present on colonoscopy images were never mapped before this study. Here, besides roundish colorectal polyps, different types of waves were also investigated using a rather broad set of possible parameters. The collection of the characteristic curves in the appendices could be used as references or extended and refined if the application deems it necessary.

For some real images, some aspects can be found by using the characteristic structures listed in this contribution. However, we did not pay attention to the fine-scale behavior, as they are not as important in the process of finding a polyp. Superpositions of different types of distributions are rather complicated, if additive and not multiplicative superstructures are studied. We suggest using wavelet-analysis or other, scale sensitive methods to separate the components of an additive superstructure.

To summarize the other findings, the following can be suggested for using structural entropy in image classification methods, especially in colorectal polyp searching cases. Instead of static tiling of the images, and using structural entropy and filling factor as two parameters of the image segment classification—although they provide valuable information about the shape of the distribution—it is more advisable to use sliding tiles and study the thus arising curves on the $S_{str}\left(\ln q\right)$ plots. We also suggest removing tilts and offsets from the image segments using simple image processing tools. We demonstrated that simple background subtraction techniques can change the characteristic curves very much without introducing extra information or losing valuable information.

Regarding the characteristic curves, we could conclude the following. The directions of the patterns do not influence the types of the characteristic curves. The size of the tiles also do not influence the results, provided that sufficiently large tile sizes are used. We suggest using larger tile sizes to achieve more stable results. In addition, for larger wavelengths, in realistic cases, the elliptical waves produce similar characteristic curves to those of the waves with straight wavefront, and they are both suitable to model the waves on the bowel walls.

## Figures and Tables

**Table 1:** The effect of the increasing of various parameters to the characteristic curves for the waves and the hemispheres in the case of off-center shift being the first parameter.

Parameter	Wave	Sphere with Shadow
General shape	periodic loops	2 hooks, M-shape
Radius	-	different position
Wavelength	different period	-
Offset	increased magnitudes	Decreased magnitudes
Tilt	broken symmetry of the shapes	smaller curves
Height ratio	-	decreased hook size
-	-	more hooks
Direction	shallower hooks	no systematic effect
Tile size	no effect for large tiles	no effect for large tiles
